# Supplementary material for: Risk Assessment and Characterization in Tuna Species of the Canary Islands According to Their Metal Content
Source: Foods. 2023 Mar 28;12(7):1438. doi: 10.3390/foods12071438 (PMC10093732; doi:10.3390/foods12071438)
Supplement: Supplementary file 1 [file foods-12-01438-s001.zip › foods-2188719-supplementary.pdf]

Supplemental data

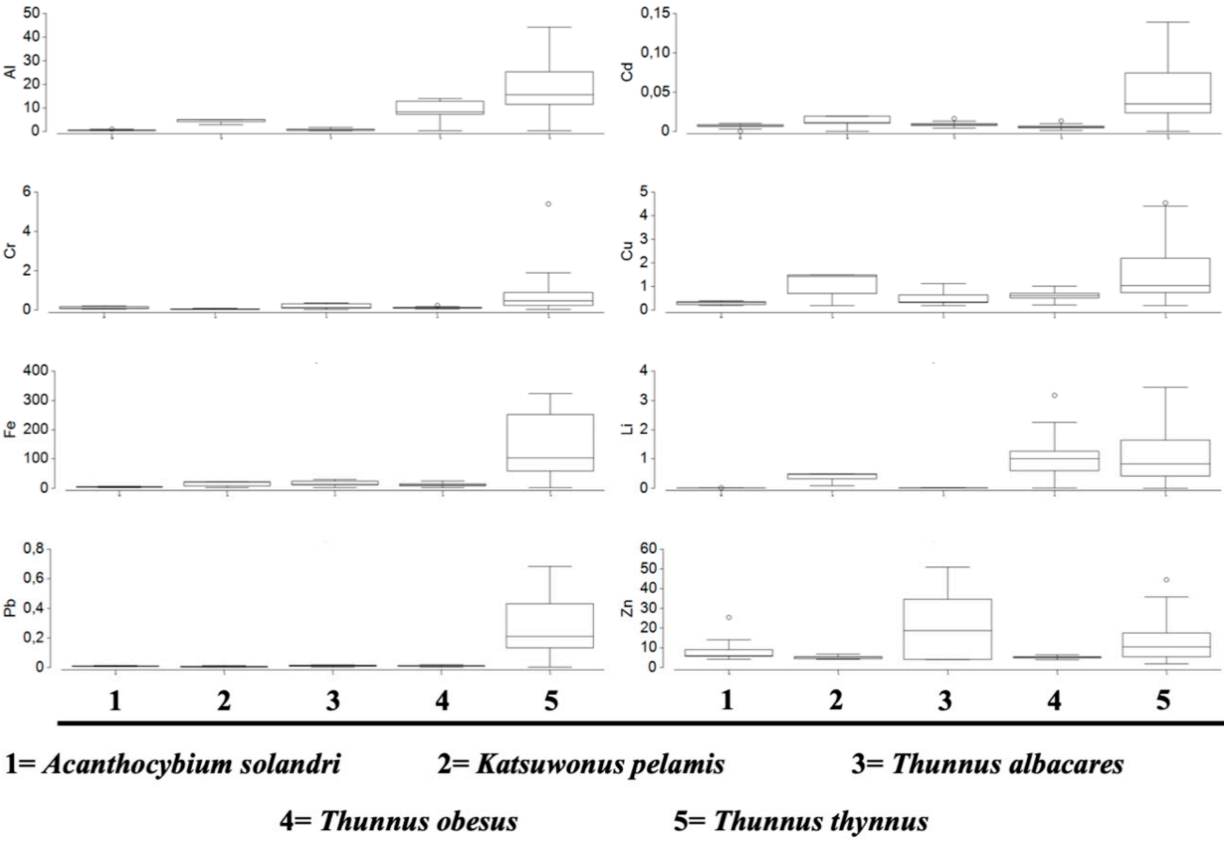

Figure S1. Box plot of metals concentrations in tuna species of the Canary Islands in mg/kg.

Table S1. Results of the pairwise test examining the significant factor "species" obtained in ANOVA for each metal.

Groups	Al	Cd	Cr	Cu	Fe	Li	Pb	Zn
Acanthocybium solandri, Katsuwonus pelamis	0.001*	0.001*	0.001*	0.001*	0.002*	0.001*	0.014*	0.076
Acanthocybium solandri, Thunnus albacares	0.329	0.001*	0.175	0.001*	0.001*	0.328	0.126	0.096
Acanthocybium solandri, Thunnus obesus	0.001*	0.888	0.432	0.001*	0.001*	0.001*	0.337	0.013*
Acanthocybium solandri, Thunnus thynnus	0.001*	0.001*	0.001*	0.001*	0.001*	0.001*	0.001*	0.276

Katsuwonus pelamis, Thunnus albacares	0.001*	0.001*	0.001*	0.001*	0.849	0.001*	0.006*	0.034*
Katsuwonus pelamis,Thunnus obesus	0.003*	0.001*	0.001*	0.001*	0.14	0.025*	0.008*	0.737
Katsuwonus pelamis, Thunnus thynnus	0,001*	0.001*	0.001*	0.656	0.002*	0.225	0.001*	0.046*
Thunnus albacares, Thunnus obesus	0.001*	0.001*	0.001*	0.065	0.212	0.001*	0.451	0.005*
Thunnus albacares, Thunnus thynnus	0.001*	0.001*	0.1	0.001*	0.001*	0.001*	0.001*	0.299
Thunnus obesus, Thunnus thynnus	0.005*	0.001*	0.001*	0.001*	0.001*	0.486	0.001*	0.016*

*$p<0.05$
